# The genome sequence of the minke whale, *Balaenoptera acutorostrata *Lacépède, 1804

**DOI:** 10.12688/wellcomeopenres.23367.1

**Published:** 2024-12-02

**Authors:** Andrew Brownlow, Nicholas J. Davison, Phillip A. Morin, Wellcome Sanger Institute Tree of Life Management, Samples and Laboratory team, Wellcome Sanger Institute Scientific Operations: Sequencing Operations, Wellcome Sanger Institute Tree of Life Core Informatics team, Tree of Life Core Informatics collective, Darwin Tree of Life Consortium

**Affiliations:** 1 University of Glasgow, Glasgow, Scotland, UK; 2 Southwest Fisheries Science Center, National Marine Fisheries Service, NOAA, La Jolla, California, USA

**Keywords:** Balaenoptera acutorostrata, minke whale, genome sequence, chromosomal, Artiodactyla

## Abstract

We present a genome assembly from an individual female *Balaenoptera acutorostrata* (the minke whale; Chordata; Mammalia; Artiodactyla; Balaenopteridae). The genome sequence has a total length of 2,772.90 megabases. Most of the assembly is scaffolded into 22 chromosomal pseudomolecules, including the X sex chromosome. The mitochondrial genome has also been assembled and is 16.42 kilobases in length.

## Species taxonomy

Eukaryota; Opisthokonta; Metazoa; Eumetazoa; Bilateria; Deuterostomia; Chordata; Craniata; Vertebrata; Gnathostomata; Teleostomi; Euteleostomi; Sarcopterygii; Dipnotetrapodomorpha; Tetrapoda; Amniota; Mammalia; Theria; Eutheria; Boreoeutheria; Laurasiatheria; Artiodactyla; Whippomorpha; Cetacea; Mysticeti; Balaenopteridae; *Balaenoptera*; *Balaenoptera acutorostrata* Lacépède, 1804 (NCBI:txid9767).

## Background

Baleen whales (Mysticeti) are the largest animals on earth, reaching up to 30 m in length and a weight of 150 metric tons. The common minke whale ( *Balaenoptera acutorostrata*) consists of two recognised subspecies, the North Atlantic minke whale ( *B. a. acutorostrata*) and the North Pacific minke whale ( *B. a. scammoni*), and one as-yet unnamed subspecies, the dwarf minke whale ( Committee on Taxonomy, 2024; NOAA Fisheries, 2024). Minke whales are the smallest and most abundant of eight recognised species in the genus *Balaenoptera*, which includes the Antarctic minke whale ( *B. bonaerensis*). The genome of the minke whale presented here is from the North Atlantic subspecies. Common minke whales are found primarily in the Northern Hemisphere, but the dwarf minke whale is seasonally sympatric with the Antarctic minke whale near portions of Africa, South America, Australia and New Zealand. Minke whales feed on a wide variety of fish species and krill in coastal and offshore waters, and along and within pack ice during summer. Reproduction is diffusely seasonal.

The common minke whale is considered to be abundant ( Perrin *et al.*, 2018) and listed as “Least Concern” by the IUCN ( Cooke, 2018) (IUCNredlist.org, consulted 5 September 2024), though it is subject to whaling by several countries, including Norway, Greenland, and Japan. Other potential threats include bycatch in fishing gear, increasing vessel traffic and pollution, and climate change.

**Figure 1.**
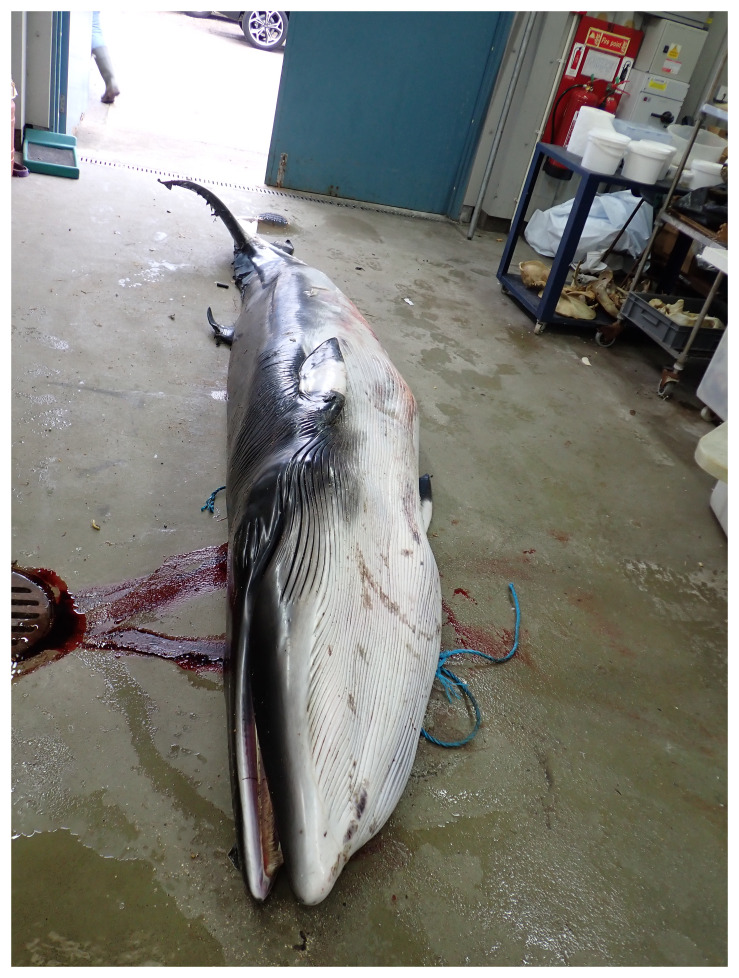
Photograph of the *Balaenoptera acutorostrata* (mBalAcu1) carcass from which samples were taken for genome sequencing.

Here we present a chromosomal-level genome sequence for *Balaenoptera acutorostrata*, based on lung tissue from the carcass of a stranded juvenile female specimen ( Figure 1) from Dalgety Bay, Fife, Scotland, UK. The genome was sequenced as part of the Cetacean Genomes Project and the Darwin Tree of Life Project.

## Genome sequence report

The genome of a juvenile *Balaenoptera acutorostrata* was sequenced using Pacific Biosciences single-molecule HiFi long reads, generating a total of 90.98 Gb (gigabases) from 10.35 million reads, providing an estimated 30-fold coverage. Primary assembly contigs were scaffolded with chromosome conformation Hi-C data, which produced 493.53 Gb from 3,268.43 million reads. Specimen and sequencing details are summarised in Table 1.

**Table 1.** Specimen and sequencing data for *Balaenoptera acutorostrata*.

Project information			
**Study title**	*Balaenoptera acutorostrata* (minke whale)		
**Umbrella BioProject**	PRJEB60647		
**Species**	*Balaenoptera acutorostrata*		
**BioSample**	SAMEA111380540		
**NCBI taxonomy ID**	9767		
Specimen information			
**Technology**	**ToLID**	**BioSample accession**	**Organism part**
**PacBio long read sequencing**	mBalAcu1	SAMEA111380548	Lung
**Hi-C sequencing**	mBalAcu1	SAMEA111380548	Lung
**RNA sequencing**	mBalAcu1	SAMEA111380548	Lung
Sequencing information			
**Platform**	**Run accession**	**Read count**	**Base count (Gb)**
**Hi-C Illumina NovaSeq 6000**	ERR11040176	3.27e+09	493.53
**PacBio Sequel IIe**	ERR11029666	2.52e+06	21.49
**PacBio Sequel IIe**	ERR11029667	2.48e+06	21.37
**PacBio Sequel IIe**	ERR11029665	2.66e+06	24.14
**PacBio Sequel IIe**	ERR11029668	2.69e+06	23.99
**RNA Illumina NovaSeq 6000**	ERR11837476	4.24e+07	6.4

**Figure 2.**
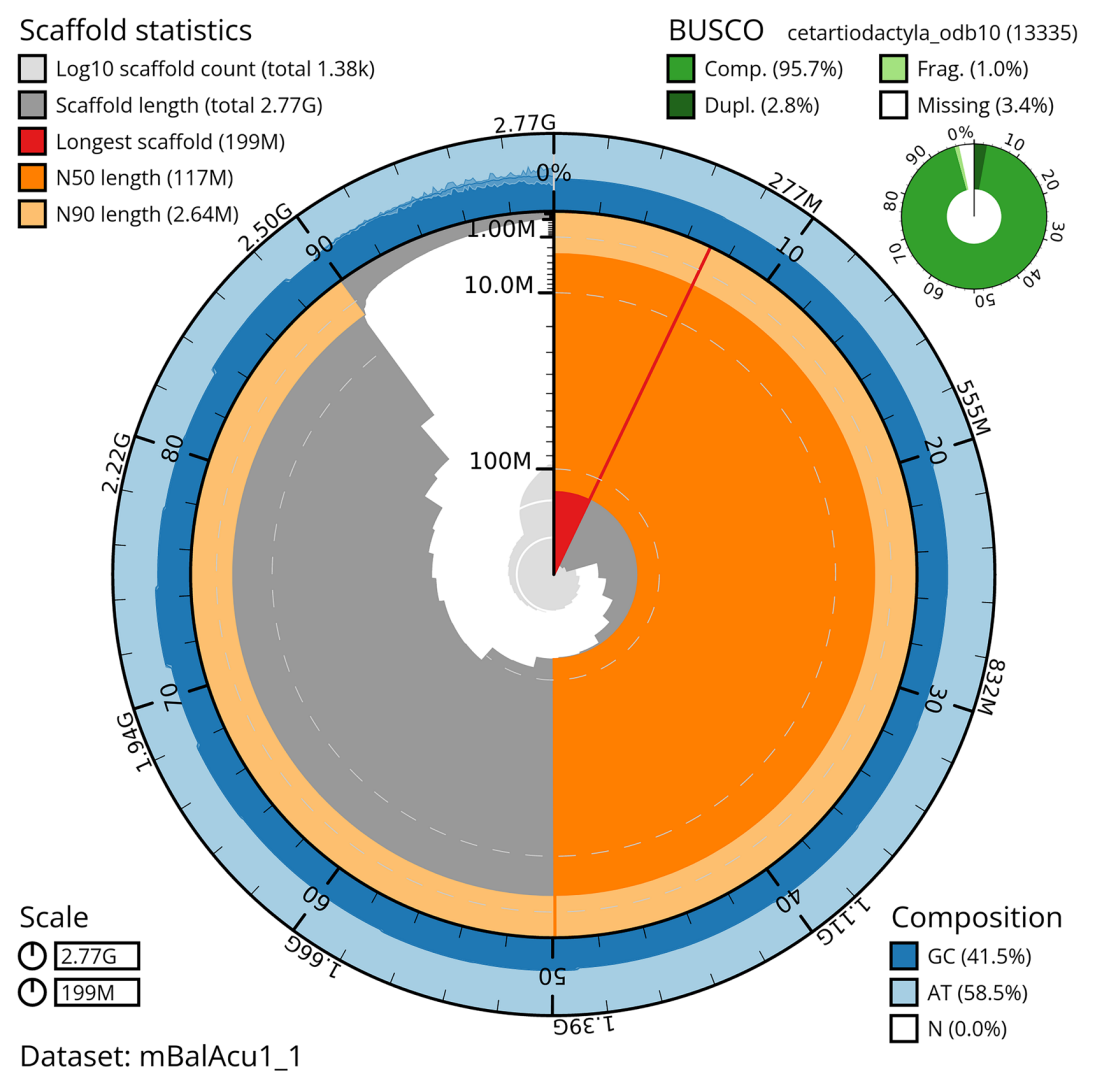
Genome assembly of *Balaenoptera acutorostrata*, mBalAcu1.1: metrics. The BlobToolKit snail plot provides an overview of assembly metrics and BUSCO gene completeness. The circumference represents the length of the whole genome sequence, and the main plot is divided into 1,000 equal-sized bins around the circumference. The outermost blue tracks display the distribution of GC, AT, and N percentages across the bins. Scaffolds are arranged clockwise from longest to shortest and are depicted in dark grey. The longest scaffold is indicated by the red arc, and the deeper orange and pale orange arcs represent the N50 and N90 lengths. A light grey spiral at the centre shows the cumulative scaffold count on a logarithmic scale. A summary of complete, fragmented, duplicated and missing BUSCO genes in the cetartiodactyla_odb10 set is shown in the top right. An interactive version of this figure is available at https://blobtoolkit.genomehubs.org/view/mBalAcu1_1/dataset/mBalAcu1_1/snail.

**Figure 3.**
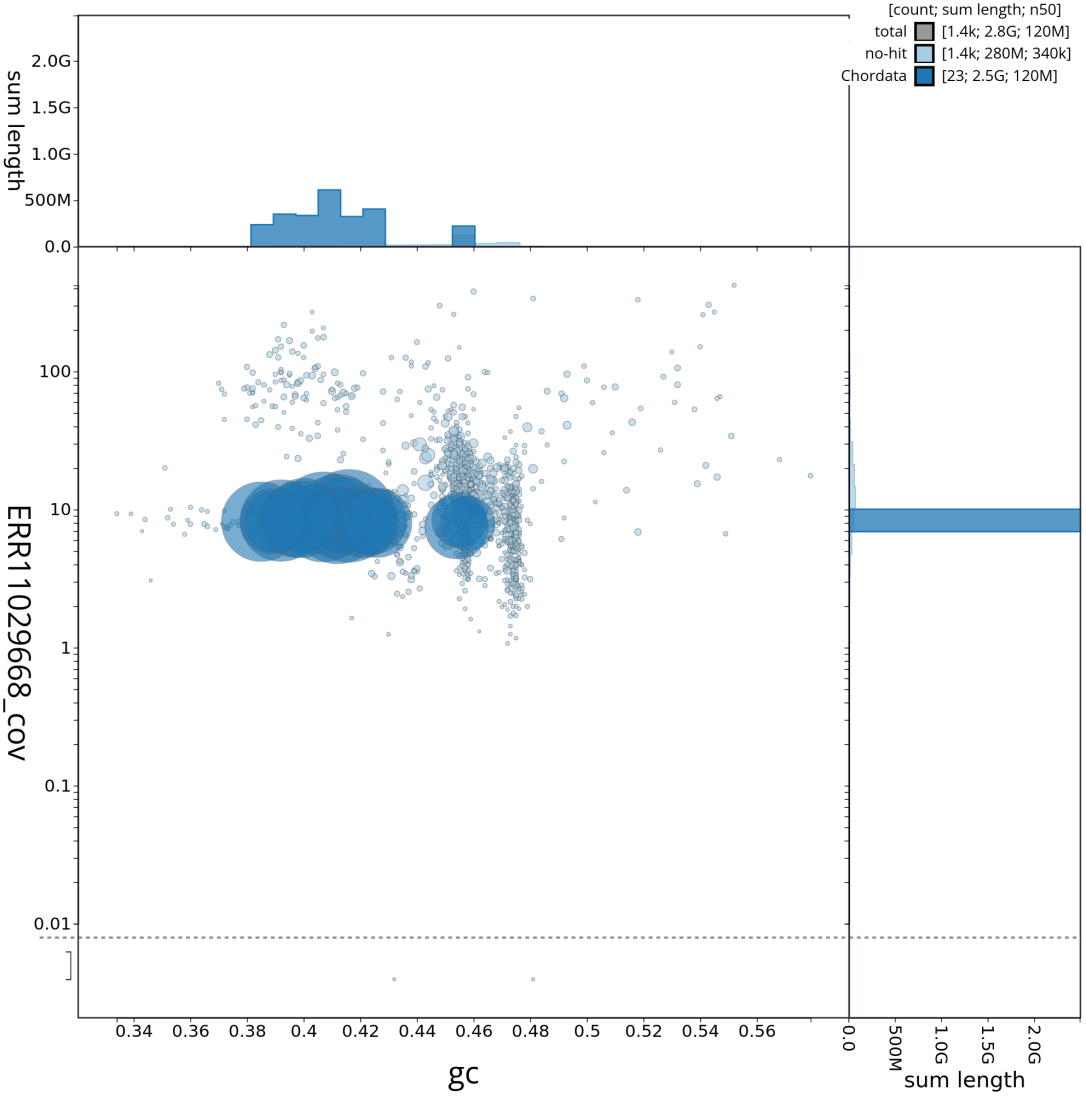
Genome assembly of *Balaenoptera acutorostrata,* mBalAcu1.1: BlobToolKit GC-coverage plot showing sequence coverage (vertical axis) and GC content (horizontal axis). The circles represent scaffolds, with the size proportional to scaffold length and the colour representing phylum membership. The histograms along the axes display the total length of sequences distributed across different levels of coverage and GC content. An interactive version of this figure is available at https://blobtoolkit.genomehubs.org/view/mBalAcu1_1/dataset/mBalAcu1_1/blob.

**Figure 4.**
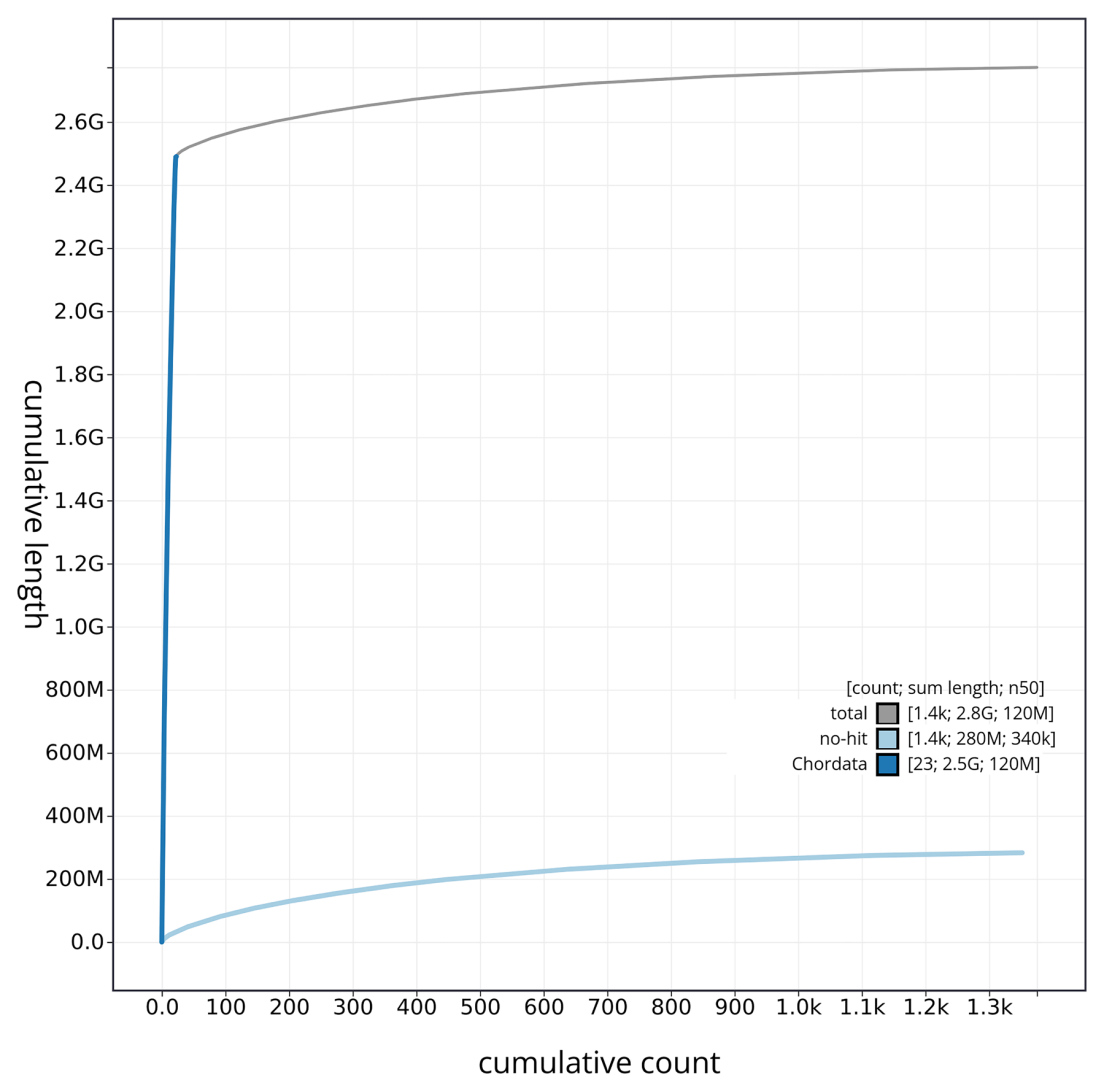
Genome assembly of *Balaenoptera acutorostrata* mBalAcu1.1: BlobToolKit cumulative sequence plot. The grey line shows cumulative length for all sequences. Coloured lines show cumulative lengths of sequences assigned to each phylum using the buscogenes taxrule. An interactive version of this figure is available at https://blobtoolkit.genomehubs.org/view/mBalAcu1_1/dataset/mBalAcu1_1/cumulative.

**Figure 5.**
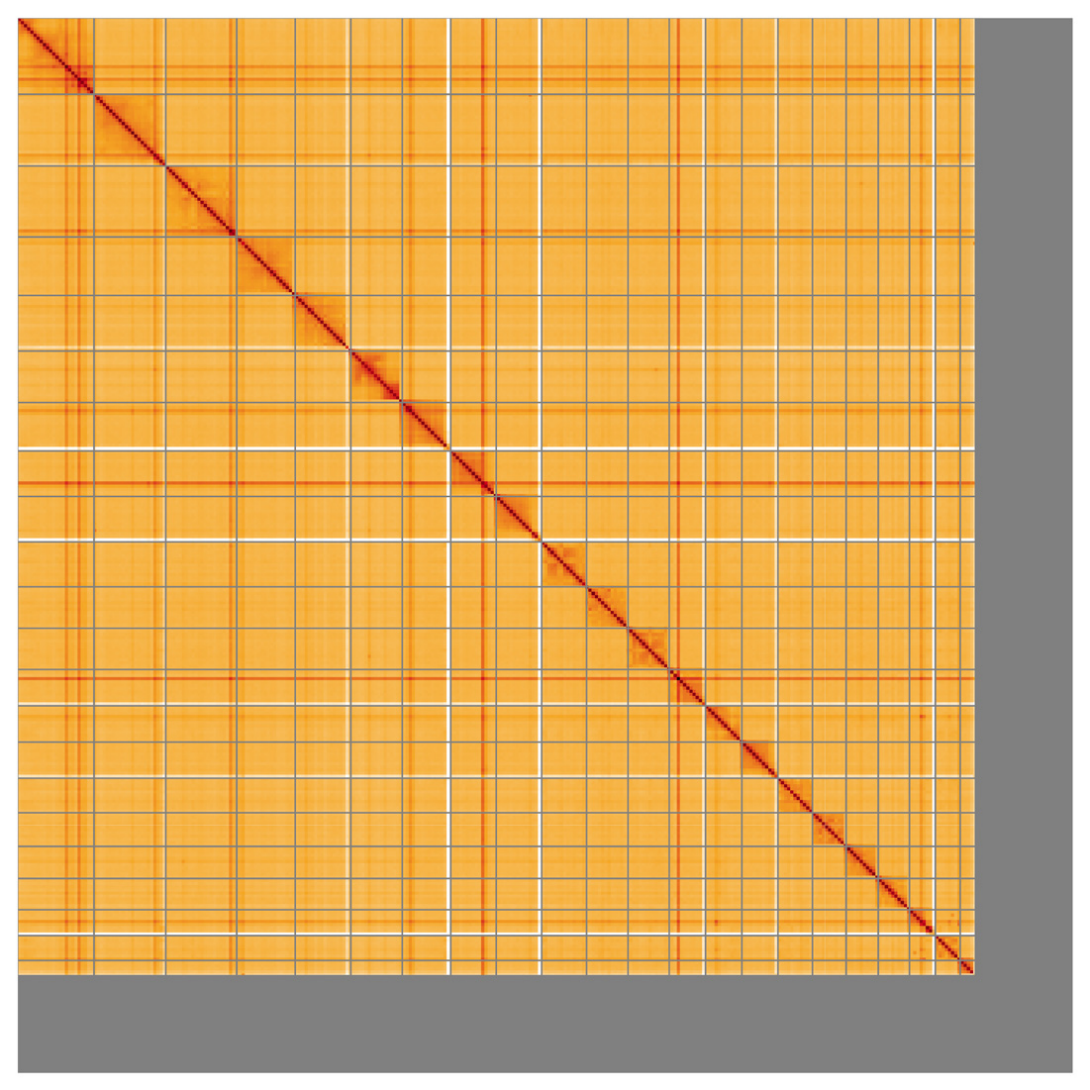
Genome assembly of *Balaenoptera acutorostrata* mBalAcu1.1: Hi-C contact map of the mBalAcu1.1 assembly, visualised using HiGlass. Chromosomes are shown in order of size from left to right and top to bottom. The darker shades indicate more frequent physical contacts between regions, while lighter areas represent fewer contacts. An interactive version of this figure may be viewed at https://genome-note-higlass.tol.sanger.ac.uk/l/?d=eVOOJg9uShC9lLe_5rxZ5Q.

Manual assembly curation corrected 150 missing joins or mis-joins, reducing the scaffold number by 5.3%, and increasing the scaffold N50 by 25.03%. The final assembly has a total length of 2,772.90 Mb in 1,374 sequence scaffolds with a scaffold N50 of 116.5 Mb ( Table 2). The total count of gaps in the scaffolds is 1,177. The snail plot in Figure 2 provides a summary of the assembly statistics, while the distribution of assembly scaffolds on GC proportion and coverage is shown in Figure 3. The cumulative assembly plot in Figure 4 shows curves for subsets of scaffolds assigned to different phyla. Most (89.88%) of the assembly sequence was assigned to 22 chromosomal-level scaffolds, representing 21 autosomes and the X sex chromosome. Chromosome-scale scaffolds confirmed by the Hi-C data are named in order of size ( Figure 5; Table 3). The X chromosome was identified based on synteny with the genome assembly of *Balaenoptera ricei* (GCA_028023285.1).

**Table 2.** Genome assembly data for *Balaenoptera acutorostrata*, mBalAcu1.1.

Genome assembly		
Assembly name	mBalAcu1.1	
Assembly accession	GCA_949987535.1	
*Accession of alternate haplotype*	*GCA_950005055.1*	
Span (Mb)	2,772.90	
Number of contigs	2,552	
Number of scaffolds	1,374	
Longest scaffold (Mb)	198.66	
Assembly metrics		Benchmark *
Contig N50 length (Mb)	3.0	*≥ 1 Mb*
Scaffold N50 length (Mb)	116.5	*= chromosome N50*
Consensus quality (QV)	62.4	*≥ 40*
*k*-mer completeness	100.0%	*≥ 95%*
BUSCO **	C:95.7%[S:92.8%,D:2.8%],F:1.0%,M:3.4%,n:13,335	*S > 90%, D < 5%*
Percentage of assembly mapped to chromosomes	89.88%	*≥ 90%*
Sex chromosomes	X	*localised homologous pairs*
Organelles	Mitochondrial genome: 16.42 kb	*complete single alleles*

* Assembly metric benchmarks are adapted from Rhie *et al.* (2021) and the Earth BioGenome Project Report on Assembly Standards September 2024. ** BUSCO scores based on the cetartiodactyla_odb10 BUSCO set using version 5.3.2. C = complete [S = single copy, D = duplicated], F = fragmented, M = missing, n = number of orthologues in comparison. A full set of BUSCO scores is available at https://blobtoolkit.genomehubs.org/view/mBalAcu1_1/dataset/mBalAcu1_1/busco.

**Table 3.** Chromosomal pseudomolecules in the genome assembly of *Balaenoptera acutorostrata*, mBalAcu1.

INSDC accession	Name	Length (Mb)	GC%
OX465350.1	1	198.66	41.5
OX465351.1	2	186.39	40.5
OX465352.1	3	183.81	41.0
OX465353.1	4	151.96	39.0
OX465354.1	5	144.37	38.5
OX465356.1	6	125.84	41.5
OX465357.1	7	118.66	40.0
OX465358.1	8	117.58	39.5
OX465359.1	9	116.51	42.0
OX465360.1	10	107.69	42.5
OX465361.1	11	107.33	41.0
OX465362.1	12	94.44	41.0
OX465363.1	13	94.05	42.5
OX465364.1	14	93.82	39.0
OX465365.1	15	90.09	45.5
OX465366.1	16	87.04	42.0
OX465367.1	17	83.27	40.0
OX465368.1	18	81.38	39.0
OX465369.1	19	67.3	45.5
OX465370.1	20	64.25	46.0
OX465371.1	21	40.0	41.0
OX465355.1	X	133.82	40.0
OX465372.1	MT	0.02	40.5

While not fully phased, the assembly deposited is of one haplotype. Contigs corresponding to the second haplotype have also been deposited. The mitochondrial genome was also assembled and can be found as a contig within the multifasta file of the genome submission.

The estimated Quality Value (QV) of the final assembly is 62.4 with *k*-mer completeness of 100.0%, and the assembly has a BUSCO v5.3.2 completeness of 95.7% (single = 92.8%, duplicated = 2.8%), using the cetartiodactyla_odb10 reference set ( *n* = 13,335). The assembly achieves the EBP reference standard of 6.7.62. Other quality metrics are given in Table 2. 

## Methods

### Sample acquisition

Samples were collected from a juvenile female *Balaenoptera acutorostrata* (specimen ID SAN00002609, ToLID mBalAcu1), found stranded in Dalgety Bay, Fife, Scotland (latitude 56.03, longitude –3.37) on 2021-04-27. The specimen was collected by Andrew Brownlow (Scottish Marine Animal Stranding Scheme University of Glasgow) and identified by Nicholas Davison (Scottish Marine Animal Stranding Scheme University of Glasgow). A sample of lung tissue was collected at necropsy and preserved by freezing at –80 °C.

### Nucleic acid extraction

The workflow for high molecular weight (HMW) DNA extraction at the Wellcome Sanger Institute (WSI) Tree of Life Core Laboratory includes a sequence of core procedures: sample preparation and homogenisation, DNA extraction, fragmentation and purification. Detailed protocols are available on protocols.io ( Denton *et al.*, 2023).

In sample preparation, the mBalAcu1 sample was weighed and dissected on dry ice ( Jay *et al.*, 2023). For sample homogenisation, lung tissue was cryogenically disrupted using the Covaris cryoPREP ^®^ Automated Dry Pulverizer ( Narváez-Gómez *et al.*, 2023). HMW DNA was extracted using the Manual MagAttract v1 protocol ( Strickland *et al.*, 2023b). DNA was sheared into an average fragment size of 12–20 kb in a Megaruptor 3 system with speed setting 30 ( Todorovic *et al.*, 2023). Sheared DNA was purified by solid-phase reversible immobilisation, using AMPure PB beads to eliminate shorter fragments and concentrate the DNA ( Strickland *et al.*, 2023a). The concentration of the sheared and purified DNA was assessed using a Nanodrop spectrophotometer and Qubit Fluorometer using the Qubit dsDNA High Sensitivity Assay kit. Fragment size distribution was evaluated by pulsed-field electrophoresis on the FemtoPulse system.

RNA was extracted from lung tissue of mBalAcu1 in the Tree of Life Laboratory at the WSI using the RNA Extraction: Automated MagMax™ *mir*Vana protocol ( do Amaral *et al.*, 2023). The RNA concentration was assessed using a Nanodrop spectrophotometer and a Qubit Fluorometer using the Qubit RNA Broad-Range Assay kit. Analysis of the integrity of the RNA was done using the Agilent RNA 6000 Pico Kit and Eukaryotic Total RNA assay.

### Hi-C preparation

Tissue from the lung of the mBalAcu1 sample was processed at the WSI Scientific Operations core, using the Arima-HiC v2 kit. In brief, frozen tissue (stored at –80 °C) was fixed, and the DNA crosslinked using a TC buffer with 22% formaldehyde. After crosslinking, the tissue was homogenised using the Diagnocine Power Masher-II and BioMasher-II tubes and pestles. Following the kit manufacturer's instructions, crosslinked DNA was digested using a restriction enzyme master mix. The 5’-overhangs were then filled in and labelled with biotinylated nucleotides and proximally ligated. An overnight incubation was carried out for enzymes to digest remaining proteins and for crosslinks to reverse. A clean up was performed with SPRIselect beads prior to library preparation.

### Library preparation and sequencing

Library preparation and sequencing were performed at the WSI Scientific Operations core. Pacific Biosciences HiFi circular consensus DNA sequencing libraries were prepared using the PacBio Express Template Preparation Kit v2.0 (Pacific Biosciences, California, USA) as per the manufacturer's instructions. The kit includes the reagents required for removal of single-strand overhangs, DNA damage repair, end repair/A-tailing, adapter ligation, and nuclease treatment. Library preparation also included a library purification step using AMPure PB beads (Pacific Biosciences, California, USA) and size selection step to remove templates shorter than 3 kb using AMPure PB modified SPRI. DNA concentration was quantified using the Qubit Fluorometer v2.0 and Qubit HS Assay Kit and the final library fragment size analysis was carried out using the Agilent Femto Pulse Automated Pulsed Field CE Instrument and 165kb gDNA and 55kb BAC analysis kit. Samples were sequenced using the Sequel IIe system (Pacific Biosciences, California, USA). The concentration of the library loaded onto the Sequel IIe was between 40–135 pM. The SMRT link software, a PacBio web-based end-to-end workflow manager, was used to set-up and monitor the run, as well as perform primary and secondary analysis of the data upon completion.

Hi-C data were generated using the Arima-HiC v2 kit. In brief, frozen tissue (–80 °C) was fixed, and the DNA crosslinked using a TC buffer containing formaldehyde. The crosslinked DNA was then digested using a restriction enzyme master mix. The 5’-overhangs were then filled in and labelled with a biotinylated nucleotide and proximally ligated. The biotinylated DNA construct was fragmented to a fragment size of 400 to 600 bp using a Covaris E220 sonicator. The DNA was then enriched, barcoded, and amplified using the NEBNext Ultra II DNA Library Prep Kit, following manufacturers’ instructions. The Hi-C sequencing was performed using paired-end sequencing with a read length of 150 bp on an Illumina NovaSeq 6000 instrument.

Poly(A) RNA-Seq libraries were constructed using the NEB Ultra II RNA Library Prep kit, following the manufacturer’s instructions. RNA sequencing was performed on the Illumina NovaSeq 6000 instrument.

### Genome assembly, curation and evaluation

***Assembly***

The HiFi reads were first assembled using Hifiasm ( Cheng *et al.*, 2021) with the --primary option. Haplotypic duplications were identified and removed using purge_dups ( Guan *et al.*, 2020). The Hi-C reads were mapped to the primary contigs using bwa-mem2 ( Vasimuddin *et al.*, 2019). The contigs were further scaffolded using the provided Hi-C data ( Rao *et al.*, 2014) in YaHS ( Zhou *et al.*, 2023) using the --break option for handling potential misassemblies. The scaffolded assemblies were evaluated using Gfastats ( Formenti *et al.*, 2022), BUSCO ( Manni *et al.*, 2021) and MERQURY.FK ( Rhie *et al.*, 2020).

The mitochondrial genome was assembled using MitoHiFi ( Uliano-Silva *et al.*, 2023), which runs MitoFinder ( Allio *et al.*, 2020) and uses these annotations to select the final mitochondrial contig and to ensure the general quality of the sequence.

***Assembly curation***

The assembly was decontaminated using the Assembly Screen for Cobionts and Contaminants (ASCC) pipeline (article in preparation). Manual curation was primarily conducted using PretextView ( Harry, 2022), with additional insights provided by JBrowse2 ( Diesh *et al.*, 2023) and HiGlass ( Kerpedjiev *et al.*, 2018). Scaffolds were visually inspected and corrected as described by Howe *et al.* (2021). Any identified contamination, missed joins, and mis-joins were corrected, and duplicate sequences were tagged and removed. The sex chromosome was identified by synteny analysis. The curation process is documented at https://gitlab.com/wtsi-grit/rapid-curation (article in preparation).

***Evaluation of the final assembly***

A Hi-C map for the final assembly was produced using bwa-mem2 ( Vasimuddin *et al.*, 2019) in the Cooler file format ( Abdennur & Mirny, 2020). To assess the assembly metrics, the *k*-mer completeness and QV consensus quality values were calculated in MERQURY.FK ( Rhie *et al.*, 2020). This work was done using the “sanger-tol/readmapping” ( Surana *et al.*, 2023a) and “sanger-tol/genomenote” ( Surana *et al.*, 2023b) pipelines. The genome readmapping pipelines were developed using the nf-core tooling ( Ewels *et al.*, 2020), use MultiQC ( Ewels *et al.*, 2016), and make extensive use of the Conda package manager, the Bioconda initiative ( Grüning *et al.*, 2018), the Biocontainers infrastructure ( da Veiga Leprevost *et al.*, 2017), and the Docker ( Merkel, 2014) and Singularity ( Kurtzer *et al.*, 2017) containerisation solutions. The genome was analysed within the BlobToolKit environment ( Challis *et al.*, 2020) and BUSCO scores ( Manni *et al.*, 2021) were calculated.

Table 4 contains a list of relevant software tool versions and sources.

**Table 4.** Software tools: versions and sources.

Software tool	Version	Source
BlobToolKit	4.2.1	https://github.com/blobtoolkit/blobtoolkit
BUSCO	5.3.2	https://gitlab.com/ezlab/busco
bwa-mem2	2.2.1	https://github.com/bwa-mem2/bwa-mem2
Cooler	0.8.11	https://github.com/open2c/cooler
Gfastats	1.3.6	https://github.com/vgl-hub/gfastats
Hifiasm	0.16.1-r375	https://github.com/chhylp123/hifiasm
HiGlass	1.11.6	https://github.com/higlass/higlass
Merqury.FK	d00d98157618f4e8d1a9190026b19b471055b22e	https://github.com/thegenemyers/MERQURY.FK
MitoHiFi	3	https://github.com/marcelauliano/MitoHiFi
PretextView	0.2	https://github.com/wtsi-hpag/PretextView
purge_dups	1.2.5	https://github.com/dfguan/purge_dups
sanger-tol/genomenote	v1.0	https://github.com/sanger-tol/genomenote
sanger-tol/readmapping	1.1.0	https://github.com/sanger-tol/readmapping/tree/1.1.0
Singularity	3.9.0	https://github.com/sylabs/singularity
YaHS	1.2a	https://github.com/c-zhou/yahs

### Wellcome Sanger Institute – Legal and Governance

The materials that have contributed to this genome note have been supplied by a Darwin Tree of Life Partner. The submission of materials by a Darwin Tree of Life Partner is subject to the **‘Darwin Tree of Life Project Sampling Code of Practice’**, which can be found in full on the Darwin Tree of Life website ( https://www.darwintreeoflife.org/project-resources). By agreeing with and signing up to the Sampling Code of Practice, the Darwin Tree of Life Partner agrees they will meet the legal and ethical requirements and standards set out within this document in respect of all samples acquired for, and supplied to, the Darwin Tree of Life Project.

Further, the Wellcome Sanger Institute employs a process whereby due diligence is carried out proportionate to the nature of the materials themselves, and the circumstances under which they have been/are to be collected and provided for use. The purpose of this is to address and mitigate any potential legal and/or ethical implications of receipt and use of the materials as part of the research project, and to ensure that in doing so we align with best practice wherever possible. The overarching areas of consideration are:

•    Ethical review of provenance and sourcing of the material

•    Legality of collection, transfer and use (national and international)

Each transfer of samples is further undertaken according to a Research Collaboration Agreement or Material Transfer Agreement entered into by the Darwin Tree of Life Partner, Genome Research Limited (operating as the Wellcome Sanger Institute), and in some circumstances other Darwin Tree of Life collaborators.

## Data Availability

European Nucleotide Archive: *Balaenoptera acutorostrata* (minke whale). Accession number PRJEB60647; https://identifiers.org/ena.embl/PRJEB60647. The genome sequence is released openly for reuse. The *Balaenoptera acutorostrata* genome sequencing initiative is part of the Darwin Tree of Life (DToL) project and the Cetacean Genomes Project (CGP). All raw sequence data and the assembly have been deposited in INSDC databases. The genome will be annotated using available RNA-Seq data and presented through the Ensembl pipeline at the European Bioinformatics Institute. Raw data and assembly accession identifiers are reported in Table 1 and Table 2. Metadata for specimens, BOLD barcode results, spectra estimates, sequencing runs, contaminants and pre-curation assembly statistics are given at https://links.tol.sanger.ac.uk/species/9767.

## Author information

Members of the Wellcome Sanger Institute Tree of Life Management, Samples and Laboratory team are listed here: https://doi.org/10.5281/zenodo.12162482.

Members of Wellcome Sanger Institute Scientific Operations: Sequencing Operations are listed here: https://doi.org/10.5281/zenodo.12165051.

Members of the Wellcome Sanger Institute Tree of Life Core Informatics team are listed here: https://doi.org/10.5281/zenodo.12160324.

Members of the Tree of Life Core Informatics collective are listed here: https://doi.org/10.5281/zenodo.12205391.

Members of the Darwin Tree of Life Consortium are listed here: https://doi.org/10.5281/zenodo.4783558.
