# Supplementary material for: The genome sequence of the minke whale, Balaenoptera acutorostrata Lacépède, 1804
Source: Wellcome Open Res. 2024 Dec 2;9:706. [Version 1] doi: 10.12688/wellcomeopenres.23367.1 (PMC11803197; doi:10.12688/wellcomeopenres.23367.1)
Supplement: Peer review history

# Reviewer report: Reviewer response for version 1

**DOI:** 10.21956/wellcomeopenres.25761.r114207

**Published:** 2024-12-12

**Authors:** Ruiqi Li

**Affiliations:** 1 University of Colorado Boulder, Boulder, Colorado, USA

The genome of the minke whale, *Balaenoptera acutorostrata *is of high-quality and it will greatly benefit the Cetacean research community. I only have a few minor questions/suggestions.

 1. Why lung tissue was choosed for genomic sequencing? 

 2. The background introduced distribution of the common minke whale ( *Balaenoptera acutorostrata*) and its relationship with other subspecies/species. Is there any hybridization between those species? Will the genome help with its taxonomy? 

 3. It would be interesting to know what questions could the genome help us to answer. See some examples in Carminati set al. 2024, and Li et al. 2023

Are sufficient details of methods and materials provided to allow replication by others?

Yes

Is the rationale for creating the dataset(s) clearly described?

Yes

Are the datasets clearly presented in a useable and accessible format?

Yes

Are the protocols appropriate and is the work technically sound?

Yes

Reviewer Expertise:

Genomics

I confirm that I have read this submission and believe that I have an appropriate level of expertise to confirm that it is of an acceptable scientific standard.

## References

[1] : Novel Megaptera novaeangliae (Humpback whale) haplotype chromosome-level reference genome. *Sci Data* .2024;11(1) : 10.1038/s41597-024-03922-9 1113 10.1038/s41597-024-03922-9 39389970 PMC11467332

[2] : The genome sequence of the giant clam, Tridacna crocea (Lamarck, 1819). *Wellcome Open Research* .2023;8: 10.12688/wellcomeopenres.20580.1 10.12688/wellcomeopenres.20580.1

[3] : The genome sequence of the giant clam, Tridacna gigas (Linnaeus, 1758). *Wellcome Open Res* .2024;9: 10.12688/wellcomeopenres.21136.1 145 10.12688/wellcomeopenres.21136.1 38800516 PMC11116938

# Reviewer report: Reviewer response for version 1

**DOI:** 10.21956/wellcomeopenres.25761.r114212

**Published:** 2024-12-05

**Authors:** Mariana F. Nery

**Affiliations:** 1 State University of Campinas, São Paulo, Brazil

The article provides a detailed presentation of the chromosomal-level genome assembly for the North Atlantic minke whale. This work represents a significant contribution to marine mammal genomics. The genome sequence is assembled to chromosomal resolution with high-quality metrics, and the method description adheres to reproducibility standards.

 The background section provides a good ecological and taxonomic context for the minke whale. However, a more explicit link between these biological or taxonomical insights and the genome assembly's potential applications could strengthen the narrative. This is my only suggestion.

Are sufficient details of methods and materials provided to allow replication by others?

Yes

Is the rationale for creating the dataset(s) clearly described?

Yes

Are the datasets clearly presented in a useable and accessible format?

Yes

Are the protocols appropriate and is the work technically sound?

Yes

Reviewer Expertise:

Molecular evolution and genomics

I confirm that I have read this submission and believe that I have an appropriate level of expertise to confirm that it is of an acceptable scientific standard.
